# Neighbor Affinity-Based Core-Attachment Method to Detect Protein Complexes in Dynamic PPI Networks

**DOI:** 10.3390/molecules22071223

**Published:** 2017-07-24

**Authors:** Xiujuan Lei, Jing Liang

**Affiliations:** School of Computer Science, Shaanxi Normal University, Xi’an 710119, China; jliang@snnu.edu.cn

**Keywords:** protein-protein interaction (PPI) network, protein complexes, neighbor affinity, core-attachment

## Abstract

Protein complexes play significant roles in cellular processes. Identifying protein complexes from protein-protein interaction (PPI) networks is an effective strategy to understand biological processes and cellular functions. A number of methods have recently been proposed to detect protein complexes. However, most of methods predict protein complexes from static PPI networks, and usually overlook the inherent dynamics and topological properties of protein complexes. In this paper, we proposed a novel method, called NABCAM (Neighbor Affinity-Based Core-Attachment Method), to identify protein complexes from dynamic PPI networks. Firstly, the centrality score of every protein is calculated. The proteins with the highest centrality scores are regarded as the seed proteins. Secondly, the seed proteins are expanded to complex cores by calculating the similarity values between the seed proteins and their neighboring proteins. Thirdly, the attachments are appended to their corresponding protein complex cores by comparing the affinity among neighbors inside the core, against that outside the core. Finally, filtering processes are carried out to obtain the final clustering result. The result in the DIP database shows that the NABCAM algorithm can predict protein complexes effectively in comparison with other state-of-the-art methods. Moreover, many protein complexes predicted by our method are biologically significant.

## 1. Introduction

With advances in high-throughput techniques, a lot of protein-protein interaction (PPI) data has been generated [1]. The emergence of large-scale PPI data has raised a hot wave of research on PPI networks in the post-genomic era. Protein interactions are important for most biological process; thus, PPI networks provide a graph of cellular mechanisms. A significant task of system biology is to explore cellular function and organization by analyzing a PPI network [2]. Almost all of the functional processes within a cell are carried out by complexes, which are formed by interaction [3]. Protein complexes participate in specific cellular functions, such as transcription of DNA, translation of mRNA and cell cycle [4]. Protein complexes can help us identify the functions of proteins [5]. The accurate prediction of complexes in PPI networks is significant for understanding the principles of cellular organization and function [6].

So far, many algorithms have been proposed to predict protein complexes from PPI networks. Bader and Hogue [7] proposed the MCODE (molecular complex detection) algorithm. Liu et al. [8] proposed the CMC (clustering based on maximal cliques) algorithm, which predicts complexes based on maximal cliques. MCL (Markov clustering) [9] was applied to identify protein complexes by simulating random walks in PPI networks. Nepusz et al. [10] presented the ClusterONE algorithm to identify protein complexes.

However, these algorithms only focus on the static PPI networks. In fact, PPI networks in cells are dynamic; they change over environment and time [11]. Therefore, the shift from static PPI networks to dynamic PPI networks is critical to identify protein complexes accurately. Wang et al. [12] injected gene expression data into static PPI networks to construct dynamic PPI networks and detect complexes. Park and Bader [13] proposed the DHAC (Dynamical Hierarchical Agglomerative Clustering) algorithm to predict temporal protein complexes from dynamic PPI networks. Ou-Yang et al. [14] presented a novel method to predict overlapping temporal protein complexes from dynamic PPI networks. Li et al. [15] presented the DPC algorithm to identify dynamic protein complexes.

In addition, Gavin et al. [16] revealed the inherent property of protein complexes. Protein complexes have a core part and an attachment part. Wu et al. [17] proposed the COACH algorithm, which is based on core attachment. Kouhsar et al. [4] used a semantic similarity measure based on Gene Ontology (GO) structure to give weights between proteins in the PPI networks. Pizzuti and Rombo [18] take advantage of genetic algorithms and six topological-based fitness functions to predict protein complexes.

To identify protein complexes accurately and biologically, researchers should pay attention to the structure properties of protein complexes predicted from dynamic PPI networks. In this paper, we proposed a novel algorithm, called NABCAM (Neighbor Affinity-Based Core-Attachment Method), to identify dynamic protein complexes. First, the centrality score of every protein is calculated. The proteins with the highest centrality scores are regarded as the seed proteins. Second, the seed proteins are expanded to complexes cores by calculating the similarity value between seed proteins and their neighbor proteins. Thirdly, the attachments are appended to their corresponding protein complex cores by comparing the affinity among neighbors inside the cluster against that outside the cluster. Finally, filtering processes are carried out. Therefore, we obtain the protein complexes set from dynamic PPI networks.

The outline of this paper is as follows. Section 2 describes some related theories and the details of our algorithms. Section 3 shows the experimental results and analysis. Section 4 concludes the paper.

## 2. Method

In this section, some relative terminologies that are used in our experiments are presented. Then, we describe the NABCAM algorithm in the following subsections.

### 2.1. Dynamic PPI Networks Construction

**Figure 1:**
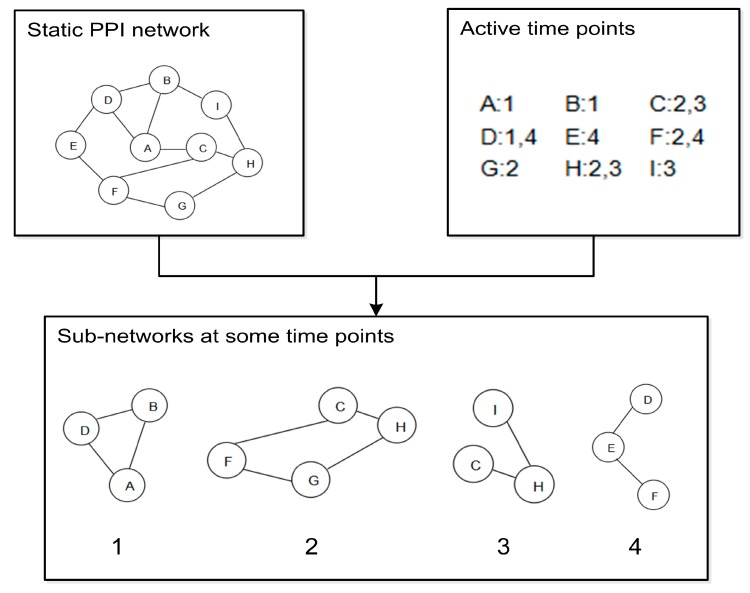
Dynamic protein-protein interaction (PPI) networks construction: (1) the subnet of time point 1; (2) the subnet of time point 2; (3) the subnet of time point 3; (4) the subnet of time point 4.

The dynamic PPI networks are constructed by integrating the static PPI data and gene expression data [19], because gene expression level and protein expression level are consistent. To identify the timestamps with high expression value of a protein, we use the three-sigma principle [12] to differentiate the active and inactive timestamps of a protein during the cellular cycle. As gene expression data has 12 timestamps, the static PPI network is divided into 12 sub-graphs, which correspond to 12 timestamps. Eventually, the dynamic PPI network is constructed. Figure 1 shows a process of dynamic PPI network construction.

### 2.2. Formation Process of Attachment

**Figure 2:**
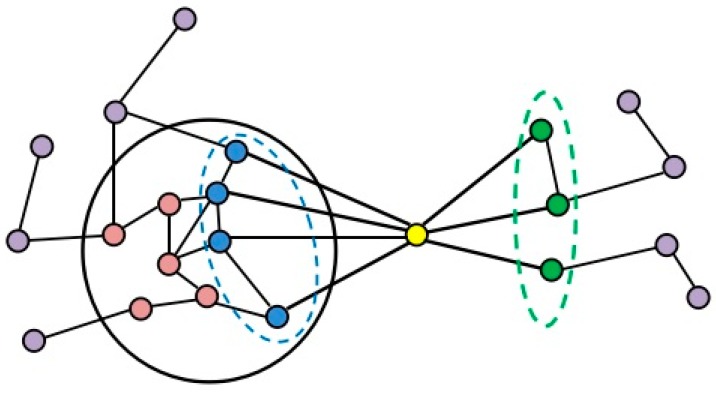
A formation process of attachment: these proteins inside the black circle constitute a complex core; the yellow protein represents a candidate neighbor protein of complex core; the blue proteins represent neighbors inside core; the green proteins represent neighbors outside core.

In this algorithm, we focus on the inherent organization of protein complexes. Based on the core-attachment structure, our algorithm identified protein complexes in dynamic PPI networks. On the formation process of attachment, we utilize the idea of neighbor affinity. As shown in Figure 2, the proteins inside the black circle constitute a complex core *c*, and the yellow protein is one of *c*’s candidate neighbor proteins to be merged, which is represented by *v*. The neighbors of *v* inside the core *c* are in the blue dotted circle, while those outside *c* are in the green dotted circle. For a protein *v*, its neighbor affinity inside core *c* and outside core *c* are defined respectively. If the *NA*(*v*) = *NAI*(*v*) − *NAO*(*v*) is more than the threshold *Tn*, the yellow protein will be merged into the core *c* as the attachment. The rest of the neighbor proteins of core *c* repeat such a process until no proteins are left to be merged. After the attachment formation, we can obtain the complexes.

### 2.3. NABCAM Algorithm

**Figure 3:**
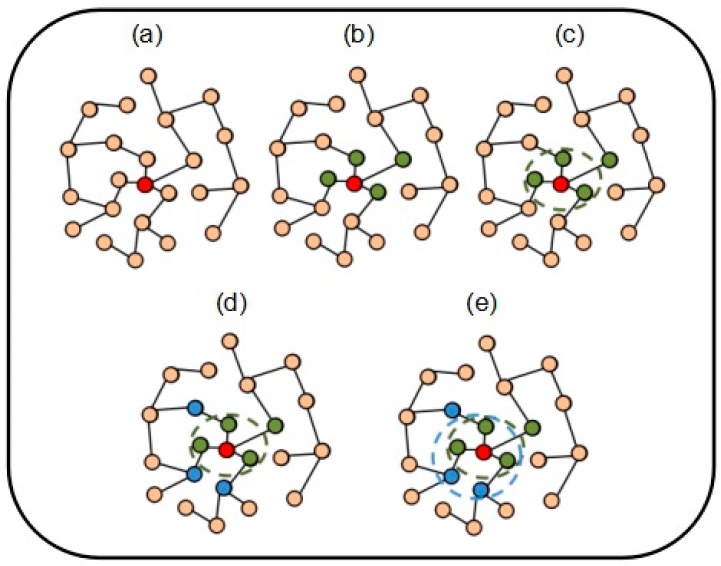
The formation process of a protein complex: (**a**) the red protein represents the seed protein; (**b**) the green proteins represent neighbor proteins of the seed protein; (**c**) these proteins inside the green dotted circle constitute a complex core; (**d**) the blue proteins represent neighbor proteins of the core; (**e**) the proteins inside the blue dotted circle constitute a protein complex.

Some analysis of protein complexes revealed the core-attachment structure of a complex [20]. In Figure 3, we visualize a formation process of a protein complex on the PPI networks to clearly describe the NABCAM algorithm.

**Figure 4:**
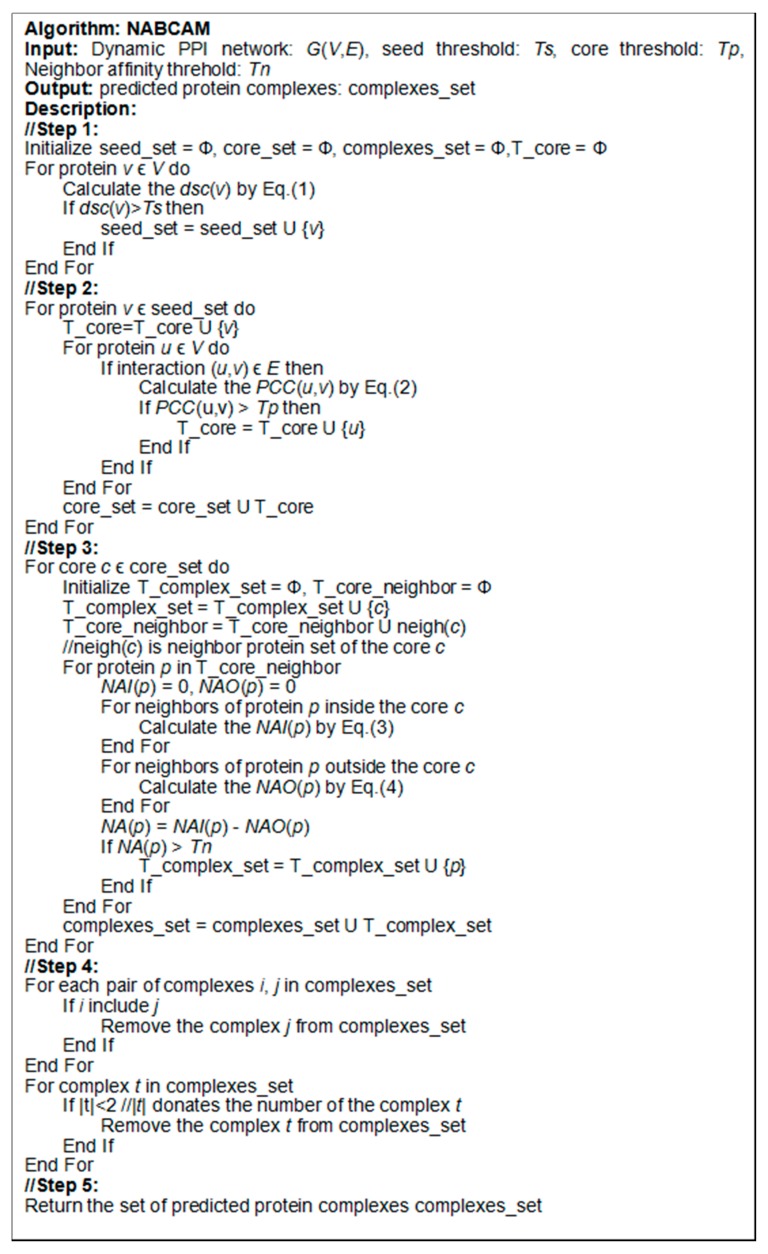
The description of the Neighbor Affinity-Based Core-Attachment Method (NABCAM) algorithm.

Based on core-attachment structure assumption, the formation process of predicted protein complexes sets involves five phases. The pseudo-code of the NABCAM algorithm is shown in Figure 4.

In the first phase (Figure 3a), the algorithm selects some seed proteins based on the dense-spread centrality score. For a protein *v* ϵ *V*, the *dsc*(*v*) is calculated by Equation (1).

(1) $$dsc(v)=dens(G_{v})*|V_{v}|$$

Considering the density and the size of the induced sub-graph of *v*, the dense-spread centrality score [21] of protein *v* is defined. For a protein *v*, its induced sub-graph is represented by $G_{v}=(V_{v},E_{v})$, where $V_{v}=\{v\cup N(v)\}$ and $E_{v}=\{(u,v)|u,v\in V_{v},(u,v)\in E\}$. The density of *G_v_* is described as following: $dens(G_{v})=2*|E_{v}|/(|V_{v}|*(|V_{v}|-1))$, where |*V_v_*| represents the number of the proteins involved in *G_v_* and |*E_v_*| represents the number of the interactions involved in *G_v_*. The protein *v* is added to the seed protein set only if *des*(*v*) > *Ts*, where *des*(*v*) is the dense-spread centrality score and *Ts* is the seed threshold. A protein *v* is discarded if it has a *des*(*v*) value less than the threshold value *Ts*. This is done for all proteins in the PPI networks. The obtained seed proteins are the primary part of the complex cores.

In the second phase (Figure 3b,c), we need to expand the seed proteins to the whole complex core. For a seed protein *v*, we compute the Pearsons correlation coefficient between seed protein *v* and its neighbor protein *u*. The *PCC* (*u*, *v*) is calculated by Equation (2).

(2) $$PCC（X,Y）=\frac{\sum \begin{array}{cc} \begin{array}{c} n\\ i=1 \end{array} & \left(x_{i}-x\prime )(y_{i}-y\prime \right) \end{array}}{\sqrt{\begin{array}{cc} \sum \begin{array}{cc} \begin{array}{c} n\\ i=1 \end{array} & {\left(x_{i}-x\prime \right)}^{2} \end{array} & \sum \begin{array}{cc} \begin{array}{c} n\\ j=1 \end{array} & {\left(y_{i}-y\prime \right)}^{2} \end{array} \end{array}}}$$

where *X* = {*x*_1_, *x*_2_, ..., *x_n_*} and *Y* = {*y*_1_, *y*_2_, ..., *y_n_*} gives the expression values of protein *X* and *Y* for *n* time points, and *x’* and *y’* give the mean of expression values of *X* and *Y,* respectively. The Pearsons correlation coefficient (PCC) is a measure of the correlation between two proteins *X* and *Y* [22]. The more similar the two proteins are, the larger their PCC value. The neighbor protein *u* is appended to the core whose seed protein is *v* only if PCC (*u*, *v*) > *Tp*, where PCC (*u*, *v*) and *Tp* is the core threshold. When all of the neighbor proteins of seed proteins are traversed over, we obtain the whole complex cores.

In the third phase (Figure 3d,e), we form a protein complex by selecting the attachments of every complex core’s peripheral information. We adopt the neighbor affinity to supplement the attachment for complex cores. For the neighbor protein *p* of the complex core *c*, we compute separately its affinity among neighbors inside and outside the core *c*, namely the values of *NAI* (*p*) and *NAO* (*p*). *NAI* (*p*) and *NAO* (*p*) are calculated by Equations (3) and (4), respectively.

(3) $$NAI(v)=\frac{\sum_{u\in NI(v)}d(u)}{\left|NI(v)\right|}$$

(4) $$NAO(v)=\frac{\sum_{u\in NO(v)}d(u)}{\left|NO(v)\right|}$$

where *v* is a neighbor protein of core *c*, and *d* (*u*) represents the number of neighbors of protein *u*. *NI* (*v*) denotes neighbors of protein *v* inside the core *c*, the number of proteins in *NI* (*v*) is represented |*NI* (*v*)|. *NO* (*v*) denotes neighbors of protein *v* outside the core *c*, the number of proteins in *NO* (*v*) is represented |*NO* (*v*)|. The difference between *NAI* (*p*) and *NAO* (*p*) is denoted by *NA* (*p*). If *NA* (*p*) > *Tn*, the protein *p* is merged into the core *c*. When all of the neighbor proteins of complex cores are traversed over, we obtain the protein complex set.

In the fourth phase, we should remove redundant protein complexes from the protein complex set. This is a significant step to purify the experimental results. For a protein complex, it may be included in other complexes. In our experiment, for the identified complexes that completely overlap with others, only one can be retained, while the others should be removed as redundant. Moreover, a predicted protein complex may only contain a protein, which also should be removed.

Finally, the NABCAM algorithm is performed on dynamic PPI networks, thereby generating the predicted protein complex set as the result.

Moreover, the seed threshold *Ts*, the core threshold *Tp* and the neighbor affinity threshold *Tn*, used in the algorithm NABCAM, decide the seed protein’s selection, complex cores formation, and protein complexes acquirement, respectively. To find the appropriate thresholds, NABCAM is run with various values of *Ts*, *Tp* and *Tn* on the DIP, MIPS, and Krogan networks, respectively. In this paper, the appropriate thresholds of *Ts*, *Tp* and *Tn* are 0.3, 0.3 and 0.

## 3. Experiments and Results

### 3.1. Experimental Datasets

In the present paper, the PPI data of *S. cerevisiae* from the DIP [23], MIPS [24] and Krogan [25] databases are used to validate the performance of NABCAM algorithm. The dynamic DIP PPI networks are 12 static PPI subnets, corresponding to 12 time points. Different subnets have different scales, as shown in Table 1. It is the same in the MIPS and Krogan datasets. The gold standard dataset of known yeast complexes is derived from CYC2008 [20], which contain 408 complexes and 1628 proteins. The biggest cluster has 81 proteins, while the smallest cluster has two proteins in the complexes of CYC2008.

### 3.2. Evaluation Criteria

To assess the performance of methods, there are three evaluation indicators: *precision*, *recall* and *f-measure* [26]. We presented the overlap score (*OS*) [12] between the predicted protein complexes and gold standard datasets. It can be defined as following Equation (5):

(5) $$OS(p,g)=\frac{|p\cap g{|}^{2}}{\left|p|\cdot |g\right|}$$

where |*p*| is the size of the identified protein complex, |*g*| is the size of the standard protein complex, and |*p*∩g| is the common protein number from the identified and gold complexes. If *OS* (*p*, *g*) ≥ *w*, we claim that *p* and *g* have been matched. In this paper, we set *w* to be equal to 0.2, which is consistent with previous articles [12].

The *precision* denotes the proportion of the predicted protein complexes perfectly matched by the standard protein complexes in the prediction of the complex. It can be defined by the following Equation (6):

(6) $$precision=\frac{N_{cp}}{\left|P\right|}$$

where |*P*| represents the number of predicted protein complexes, and *N_cp_* indicates that the number of the predicted complexes perfectly matched by the known protein complexes. The higher *precision* is, the more accurate the algorithm is.

The *recall* indicates the proportion of the known protein complexes perfectly matched by the predicted protein complexes in the standard of the protein complex. It can be defined by the following Equation (7):

(7) $$recall=\frac{N_{cb}}{\left|B\right|}$$

where |*B*| represents the number of known protein complexes, and *N_cb_* indicates the number of standard protein complexes perfectly matched by the predicted protein complexes. The higher *recall* is, the more accurate the algorithm is for predicting protein complexes.

The *precision* and *recall* describe the effectiveness of the algorithm from different aspects. In order to consider these indicators synthetically, the *f-measure* is defined as the harmonic mean of *precision* and *recall*, which can access the overall performance of a method. It is defined by the following Equation (8):

(8) $$f-measure=\frac{2\times precision\times recall}{precision+recall}$$

From the formula of harmonic mean, we can see that *precision* and *f-measure* have a relationship of positive correlation. Similarly, *recall* and *f-measure* also have a relationship of positive correlation. 

In order to further validate the biological significance of protein complexes, we need to carry out the functional enrichment analysis by using the *p-value* [27] formulated through the following Equation (9):

(9) $$p-value=\sum_{i=m}^{n}\frac{\left(\begin{array}{c} M\\ i \end{array}\right)\left(\begin{array}{c} N-M\\ n-i \end{array}\right)}{\left(\begin{array}{c} N\\ n \end{array}\right)}$$

where *N* is the number of proteins in the PPI network, *M* is the number of proteins in a GO term, and *n* is the number of proteins that are annotated with the same GO term. Generally, the smaller the *p-value* of a protein complex, the stronger the biological significance of the complex processes will be. In this paper, a detected complex is considered to be significant if its *p-value* is less than 0.01.

### 3.3. Comparison with Known Complexes

**Figure 5:**
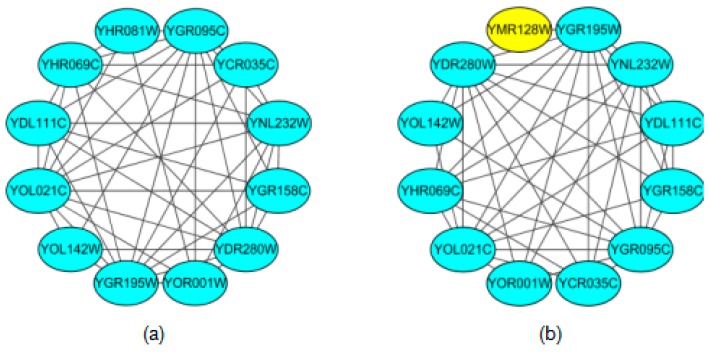
Visualization of a protein complex: (**a**) standard complex; (**b**) identified complex: the yellow protein represents the wrong protein; the blue proteins represent correct proteins.

In this section, the predicted protein complexes are compared with the standard protein complexes. In Figure 5, we visualize a protein complex to clearly show the performance of the NABCAM algorithm. In Figure 5a, there are 12 proteins in this standard complex. In Figure 5b, there are 12 proteins in the complex we identified. Our algorithm predicted 11 proteins accurately. The protein YHR081W is the missed protein, and the protein YMR128W is detected falsely.

### 3.4. Comparison Based on Precision, Recall and F-Measure

**Figure 6:**
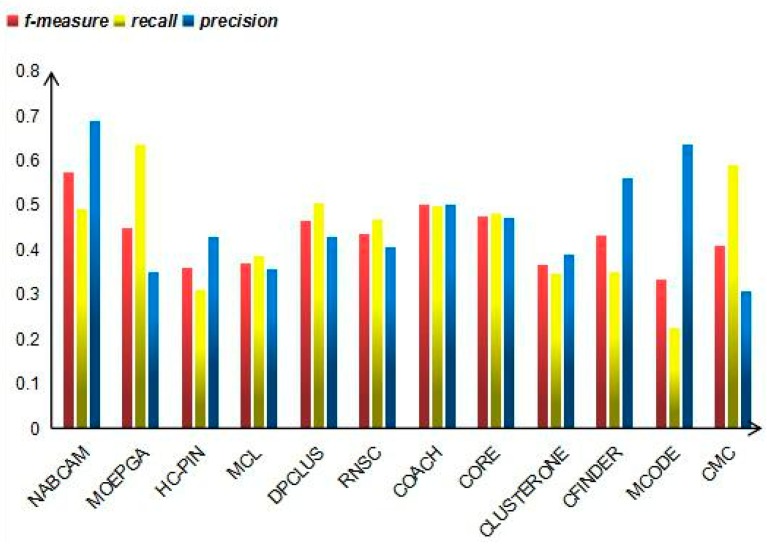
*Precision*, *recall* and *f-measure* values of various algorithms on the DIP dataset.

As shown in Figure 6, we compared our algorithm on dynamic DIP PPI networks with the following state-of-the-art protein complex prediction algorithms: MOEPGA [28], HC-PIN [29], MCL [30], DPClus [31], RNSC [23], COACH [17], CORE [32], ClusterOne [10], CFinder [33], MCODE [7], and CMC [8]. When using the dynamic DIP PPI networks, the NABCAM method achieves *precision*, *recall* and *f-measure* values of 0.6903, 0.4917 and 0.5743, respectively. It is obvious that the *precision* value of our method is much more excellent than other prediction methods. Compared with other methods, our algorithm’s *recall* value is a little lower than the *recall* values of MOEPGA, DPClus, COACH and CMC. However, the *f-measure* is higher for the NABCAM algorithm than its counterpart methods. The other methods MOEPGA, HC-PIN, MCL, DPClus, RNSC, COACH, CORE, ClusterOne, CFinder, MCODE and CMC achieved *f-measure* values of 0.4510, 0.3600, 0.3717, 0.4653, 0.4359, 0.5019, 0.4766, 0.3680, 0.4331, 0.3342 and 0.4100.

**Figure 7:**
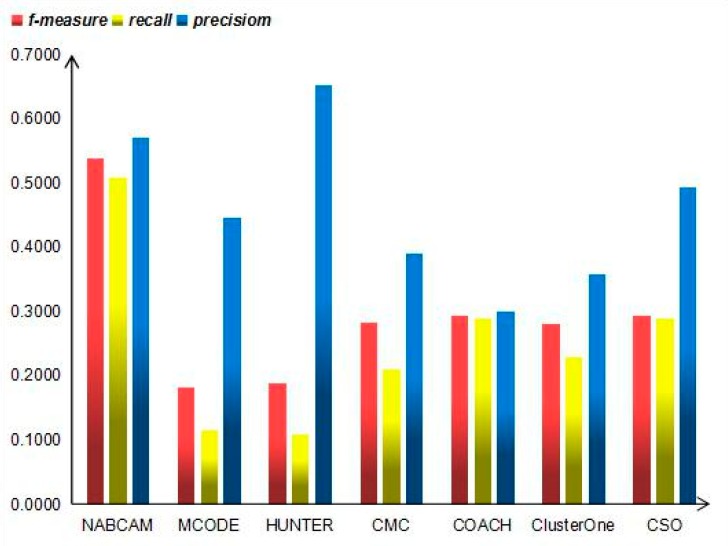
*Precision*, *recall* and *f-measure* values of various algorithms on the MIPS dataset.

**Figure 8:**
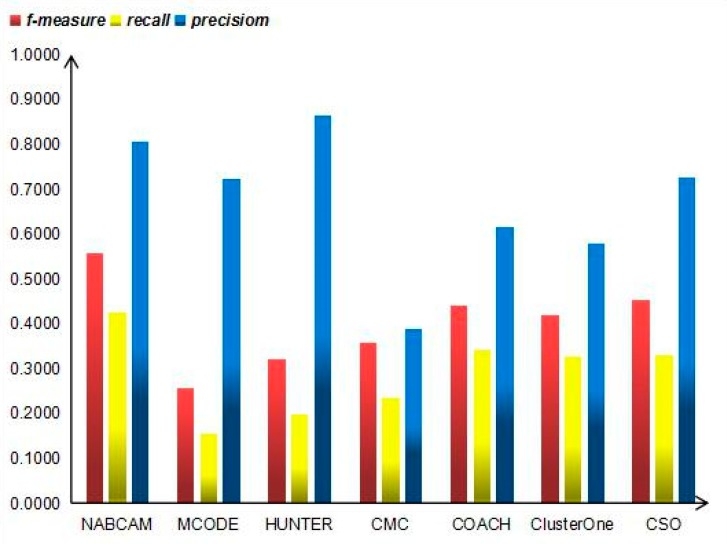
*Precision*, *recall* and *f-measure* values of various algorithms on the Krogan dataset.

Moreover, we also compare our method with the following established leading protein complex prediction methods: CSO [34], ClusterOne [10], COACH [17], CMC [8], HUNTER [35], and MCODE [7] in terms of *precision*, *recall* and *f-measure* in the MIPS and Krogran datasets, respectively, as shown in Figure 7 and Figure 8. As shown in Figure 7, our method achieves the highest *f-measure* of 0.5382, *recall* of 0.5094, and *precision* of 0.5706 in MIPS dataset, which obviously outperforms other methods. In Figure 8, it can be seen that our method achieves the highest *f-measure* of 0.5575, *recall* of 0.4259 and *precision* of 0.8068 in the Krogan dataset, which obviously outperforms other methods.

### 3.5. Comparison Based on Gene Ontology (GO) Semantic

A complex is considered significant when its *p-value* is less than 0.01. In this experiment, we use the tool GO::TermFinder [21] to calculate the *p-value* of identified complexes whose size is greater than two.

Table 2 lists the number and percentage of the predicted protein complexes whose *p-value* is in the range of <10^−^^15^, [10^−^^15^, 10^−^^10^), [10^−^^10^, 10^−^^5^), [10^−^^5^, 0.01), ≥0.01. Table 2 shows the comparison of the functional enrichment of complexes identified by NABCAM, MCL, CORE and ClusterOne, in the DIP, MIPS and Krogan datasets. As shown in Table 2, we can obtain the number of predicted protein complexes by different methods on different datasets. The percentage and the amount of the predicted protein complexes with *p-values* greater than 0.01 fall into corresponding intervals. We can see from Table 2 that our algorithm outperforms the MCL, CORE and ClusterOne algorithms. In the DIP dataset, the percentage of complexes whose *p-value* is greater than 0.01 in predicted complexes by the NABCAM algorithm is the smallest. So, most of the predicted protein complexes by the NABCAM algorithm are significant. Similarly, we can obtain results on the MIPS and Krogan datasets. The results illustrate that the NABCAM algorithm is competent at identifying significant protein complexes in PPI networks.

To further reveal the biological significance of predicted complexes, five identified protein complexes with different datasets are presented in Table 3, which lists the *p-value* of protein complexes, cluster frequency, and the Gene Ontology term.

## 4. Conclusions

In the post-genomic era, it’s significant to understand the topological organization of PPI networks, predict protein complexes and discover the functions of proteins. For the sake of these goals, a number of prediction algorithms have been proposed. In this paper, we proposed a novel algorithm, NABCAM, for the computational prediction of protein complexes on dynamic PPI networks. In the NABCAM method, first, some proteins with high dense-spread centrality scores are regarded as seed proteins. Second, the seed proteins are expanded to complexes cores by calculating the similarity value between the seed protein and its neighbor protein. And then the attachments are appended to their corresponding protein complex cores by comparing the affinity among neighbors inside the cluster against that outside the cluster. Our method considers the dynamic properties of PPI networks and the inherent organization of complexes.

Our algorithm is evaluated and analyzed by comparing it with other state-of-the-art algorithms in terms of *precision*, *recall* and *f-measure*. Experimental results show that the NABCAM algorithm has a better performance than other methods. Moreover, a number of protein complexes with strong biological significance are identified from dynamic PPI networks by our algorithm. In the future, we will attempt to apply our algorithm to other organisms.

## Figures and Tables

**Table 1:** The number of proteins and interactions in each subnet of different PPI networks.

Data	Timestamp	1	2	3	4	5	6	7	8	9	10	11	12
DIP	Proteins	797	941	796	623	601	530	493	944	1090	592	661	461
	Interactions	981	1444	1188	745	750	646	573	1705	2185	856	974	526
MIPS	Proteins	737	897	781	583	570	531	470	839	1014	523	616	402
	Interactions	1097	1443	1183	754	684	642	504	1238	1637	878	1207	700
Krogan	Proteins	336	379	320	256	206	189	202	580	626	304	330	250
	Interactions	334	464	331	234	210	184	213	1025	1081	314	373	258

**Table 2:** Functional enrichment analysis of complexes detected on different datasets.

Data	Algorithm	PC	<10^−15^	[10^−15^, 10^−10^)	[10^−10^, 10^−5^)	[10^−5^, 0.01)	≥0.01
DIP	NABCAM	1702	136 (7.99%)	230 (13.51%)	820 (48.18%)	343 (20.15%)	173 (10.16%)
	MCL	1053	19 (1.80%)	47 (4.46%)	183 (17.38%)	362 (34.38%)	442 (41.98%)
	CORE	344	1 (0.29%)	3 (0.87%)	78 (22.67%)	114 (33.14%)	148 (43.02%)
	ClusterOne	574	21 (3.66%)	52 (9.06%)	177 (30.84%)	184 (32.06%)	140 (24.39%)
MIPS	NABCAM	966	30 (3.10%)	70 (7.25%)	332 (34.37%)	333 (34.47%)	201 (20.81%)
	MCL	606	5 (0.83%)	13 (2.15%)	94 (15.51%)	220 (36.30%)	274 (45.21%)
	CORE	340	0 (0.00%)	4 (1.18%)	65 (19.12%)	107 (31.47%)	164 (48.24%)
	ClusterOne	372	7 (1.88%)	16 (4.30%)	117 (31.45%)	126 (33.87%)	106 (28.49%)
Krogan	NABCAM	587	75 (12.78%)	75 (12.78%)	304 (51.79%)	108 (18.39%)	25 (4.26%)
	MCL	403	16 (3.97%)	43 (10.67%)	103 (25.56%)	119 (29.53%)	122 (30.27%)
	CORE	255	3 (1.18%)	10 (3.92%)	60 (23.53%)	102 (40.00%)	80 (31.37%)
	ClusterOne	399	13 (3.26%)	43 (10.78%)	98 (24.56%)	120(30.08%)	125 (31.33%)

**Table 3:** Predicted protein complexes with small *p-values* on different datasets.

Data	ID	*p-Value*	Cluster Frequency	Gene Ontology Term
DIP	1	2.45 × 10^−47^	30 out of 34 genes, 88.2%	ribosomal small subunit biogenesis
	2	4.48 × 10^−38^	22 out of 23 genes, 95.7%	mRNA splicing, via spliceosome
	3	1.41 × 10^−37^	21 out of 21 genes, 100.0%	mRNA splicing, via spliceosome
	4	3.88 × 10^−36^	22 out of 23 genes, 95.7%	ribosomal small subunit biogenesis
	5	1.45 × 10^−33^	12 out of 12 genes, 100.0%	polyadenylation-dependent snoRNA 3′-end processing
MIPS	1	9.62 × 10^−27^	16 out of 18 genes, 88.9%	ribosomal large subunit biogenesis
	2	1.15 × 10^−25^	18 out of 25 genes, 72.0%	mitotic sister chromatid segregation
	3	1.15 × 10^−23^	16 out of 17 genes, 94.1%	mitotic nuclear division
	4	4.02 × 10^−23^	14 out of 16 genes, 87.5%	ribosomal large subunit biogenesis
	5	2.69 × 10^−22^	16 out of 23 genes, 69.6%	mitotic sister chromatid segregation
Krogan	1	1.36 × 10^−34^	17 out of 18 genes, 94.4%	ncRNA transcription
	2	3.69 × 10^−34^	13 out of 15 genes, 86.7%	tRNA catabolic process
	3	2.49 × 10^−33^	13 out of 14 genes, 92.9%	chromatin disassembly
	4	3.17 × 10^−32^	13 out of 16 genes, 81.2%	exonucleolytic trimming to generate mature 3′-end of 5.8S rRNA from tricistronic rRNA transcript (SSU-rRNA, 5.8S rRNA, LSU-rRNA)
	5	5.79 × 10^−32^	18 out of 18 genes, 100.0%	mRNA splicing, via spliceosome
